# Looking back, looking forward

**DOI:** 10.5195/jmla.2023.1711

**Published:** 2023-04-21

**Authors:** Jill T. Boruff, Michelle Kraft

**Affiliations:** 1 jill.boruff@mcgill.ca, Co-Editor in Chief, *Journal of the Medical Library Association*, Associate Librarian, Schulich Library of Physical Sciences, Life Sciences, and Engineering, McGill University, Montreal, QC, Canada.; 2 kraftm@ccf.org, Co-Editor in Chief, *Journal of the Medical Library Association*, Medical Library Director, Cleveland Clinic Foundation, Cleveland, OH, United States.

## Abstract

The second half of 2022 was a time of much change at the *Journal of the Medical Library Association* (*JMLA*). We hope to lead this journal with transparency, and in this spirit, we wanted to give you an overview of what we have done since we were appointed as co-editors in chief (co-EICs) in June 2022.

## FROM SUBMISSION TO PUBLICATION

We made the decision to combine the January and April issue for pragmatic reasons. This combined issue has fewer items than a typical *JMLA* issue, but it is not from a lack of compelling manuscript submissions. We have many interesting submissions currently moving through the process and continue to receive submissions every week. Typically, each of our editors has a number of manuscripts in different stages of the process at any one time. This allows for manuscripts to be ready for publication in future issues in a gradual manner. However, many events converged to disrupt this pipeline of submitted manuscripts moving to publication.

With the departure of our copy and production editor in May 2022, any author with a manuscript accepted from that time forward had to wait until a new copy and production editor was hired to move along in the process. When our new copy and production editor Katie Arnold started in September, she had two issues worth of manuscripts to prepare for publication, while at the same time learning our journal management software and our standard operating procedures. Since the authors of these manuscripts had already waited months to have their manuscripts published, we chose not to hold any of them over for the January 2023 issue and published them in the July and October 2022 issues.

During this waiting period, with such a large publication backlog, the senior editorial team slowed down on processing new submissions and focused on helping us, the co-EICs, understand our new roles. By slowing down the movement of submissions into the pipeline, we created the awkward situation of having few manuscripts ready for copyediting for the January and April 2023 issues. The good news is that we are slowly getting back on track, and future issues will gradually resume their typical size. By combining the January and April 2023 issues, we are able to focus all our attention on producing a July 2023 issue on time.

## PEER REVIEWERS

Another challenge for keeping submissions moving through the pipeline is finding peer reviewers. We have all taken stock of our time and work-life balance in recent years, and as a result, peer reviewers are more frequently declining requests to review. We mention this fact not to shame people into taking on extra work; quite the opposite. It is to remind us all—editors, authors, and readers—that manuscripts might move a bit more slowly to publication. We are gradually implementing the ideas mentioned in previous editorials to enlarge our peer reviewer pool [1, 2].

If you are interested in peer reviewing, please express your interest to us by emailing the MLA publication director at stuart.hales@mlahq.org.

## STRIVING FOR DIVERSITY EQUITY AND INCLUSION

At the same time that we manage the journal production, we continue the work of diversity, equity, and inclusion by “providing more equitable opportunities for authors, reviewers, and editorial team members.”[2] Last year's call for Editorial Board Members reflected our commitment to equity to bring a greater diversity of perspectives and life experiences to the Editorial Board. [3] We will continue to seek individuals with diverse personal identities, professional roles, workplaces, geographies and strongly encourage individuals who identify as being from underrepresent groups within LIS publishing to apply this coming year to be on the Editorial Board. The equity working group meets regularly and is planning future ongoing Diversity, Equity, and Inclusion training for the editorial team.

We have also worked with the MLA Style Guide Task Force, who is responsible for creating an updated guide for *JMLA* and other MLA publications. Particular attention has been paid on clear guidance for terms on race, ethnicity, and disability. We hope to have the new style guide available by this summer.

## BIG IDEAS

We are excited for 2023 and all the ideas and plans we have for *JMLA*. With the journal publication schedule back on track, we hope that we can finally tackle other projects in addition to the day-to-day journal production. The January/April 2023 issue is the first issue without a print format, and we now have made the entire issue available for download in a single PDF.
